# Ventricular Dysfunction in Obese and Nonobese Rats with Metabolic Syndrome

**DOI:** 10.1155/2022/9321445

**Published:** 2022-02-22

**Authors:** Julian Torres-Jacome, Brian Sabino Ortiz-Fuentes, Daniela Bernabe-Sanchez, Benjamin Lopez-Silva, Myrian Velasco, Martha Lucia Ita-Amador, Alondra Albarado-Ibañez

**Affiliations:** ^1^Laboratorio de Fisiopatología Cardiovascular, Instituto de Ciencias, Benemérita Universidad Autónoma de Puebla, Puebla, Mexico; ^2^Neuroscience Division, Instituto de Fisiología Celular, Department of Cognitive Neuroscience, Universidad Nacional Autónoma de México, México City, Mexico; ^3^Laboratorio de Fisiopatología Cardiovascular, Complejo Nororiental, Benemérita Universidad Autónoma de Puebla, Puebla, Mexico; ^4^Laboratorio de Aplicaciones Biotecnológicas, Instituto de Ciencias, Benemérita Universidad Autónoma de Puebla, Puebla, Mexico

## Abstract

Obesity and dyslipidemias are both signs of metabolic syndrome, usually associated with ventricular arrhythmias. Here, we tried to identify cardiac electrical alteration and biomarkers in nonobese rats with metabolic syndrome (MetS), and these findings might lead to more lethal arrhythmias than obese animals. The MetS model was developed in Wistar rats with high-sucrose diet (20%), and after twenty-eight weeks were obtained two subgroups: obese (OMetS) and nonobese (NOMetS). The electrocardiogram was used to measure the ventricular arrhythmias and changes in the heart rate variability. Also, we measured ventricular hypertrophy and its relationship with electrical activity alterations of both ventricles, using micro-electrode and voltage clamp techniques. Also, we observed alterations in the contraction force of ventricles where a transducer was used to record mechanical and electrical papillary muscle, simultaneously. Despite both subgroups presenting long QT syndrome (0.66 ± 0.05 and 0.66 ± 0.07 ms with respect to the control 0.55 ± 0.1 ms), the changes in the heart rate variability were present only in OMetS, while the NOMetS subgroup presented changes in QT interval variability (NOMetS SD = 1.8, SD2 = 2.8; SD1/SD2 = 0.75). Also, the NOMetS revealed tachycardia (10%; *p* < 0.05) with changes in action potential duration (63% in the right papillary and 50% in the left papillary) in the ventricular papillary which are correlated with certain alterations in the potassium currents and the force of contraction. The OMetS showed an increase in action potential duration and the force of contraction in both ventricles, which are explained as bradycardia. Our results revealed lethal arrhythmias in both MetS subgroups, irrespectively of the presence of obesity. Consequently, the NOMetS showed mechanical-electrical alterations regarding ventricle hypertrophy that should be at the NOMetS, leading to an increase of CV mortality.

## 1. Introduction

The MetS is known as a cluster of risk factors (impaired fasting glucose, insulin resistance, hypertension, dyslipidemias, and central obesity) [1–3] for type 2 diabetes mellitus and cardiovascular diseases, which occur together more often than by chance alone [4], that are associated with excess morbidity or/and mortality in humans [1, 5].

Obesity is a global epidemic for children [6] and adults, increasing the risk for cardiovascular morbidity and mortality, and the fact that obese along with overweight people are more prone to develop hypertension, hyperinsulinemia, dyslipidemias, and glucose homeostasis alteration [7–9].

The risk of development of cardiovascular diseases such as congestive heart failure, myocardial infarction, atrial fibrillation, and dilated cardiomyopathy is also increased [10, 11]. Generally, cardiomyopathy is commonly associated with electrocardiographic abnormalities [12], including impaired cardiac contractile [13]. Moreover, the long QT syndrome is related to alterations in ventricular electrical repolarization [14], and QT interval dispersion of ECG [15]. The alterations in beat-to-beat cardiac are clinically associated with cardiovascular risks [16]. One way of prognosis and diagnosis of cardiac diseases is to determine the heart rate variability (HRV) with the interval electrocardiogram (ECG) analysis [17]. On the other hand, the HRV is a noninvasive method that allows prognostic left ventricular dysfunction [18] and prolongation of QT intervals which are considered a factor risk to ventricular arrhythmias like Torsade de Pointes [17, 18]. Moreover, HRV is used to assess heart's health status at the prognosis, diagnosis of diabetes mellitus, hypertension, MetS, and obesity [19–21]. The relationship between lethal ventricular arrhythmias, cardiomyopathies, and LV dysfunction (associated with dyslipidemias and visceral fat) has been poorly identified and discussed in current literature [22, 23].

In this research, the MetS model gives us some information about the cardiomyopathies and lethal arrhythmias in obese and nonobese animal models. Our hypotheses is that the NOMetS could show higher probability of more lethal arrhythmias than in OMetS, the NOMetS is not usually considered as a cardiac critical problem due to clinically confuse it the obesity is condition to dyslipidemias and cardiac electrical alterations. Consequently, we suggest that the quantification of blood plasma biochemistry and electrocardiogram analysis allowed us to know more about arrhythmias more than waist measure in subjects with MetS.

## 2. Material and Methods

### 2.1. Animal Model

All animal procedures were performed according to the International Guiding Principles for Biomedical Research Involving Animals Council for the International Organization of Medical Science 2010, including the Animal Ethics Committee of the Internal Council and the Animal Care Committee of the Instituto de Fisiología Celular at the Universidad Nacional Autónoma de México. Twenty young adult male Wistar rodents (250-280 g) were kept in a 12 h light/dark cycle. MetS was induced by feeding with standard rat chow, composed by laboratory rodent diet (LABDIET 5001) with 28.5% of protein, 13.5% of fat, and 58% of carbohydrates [24]. Ad libitum tap water was provided to the control group and 20% (*w/v*) of sucrose solution to the experimental group for a twenty-eight-week treatment. The animals were anesthetized with an intraperitoneal sodium pentobarbital injection (40 mg/kg) [25]. The following measures were taken by abdominal circumference, epididymal fat, body weight, and body length. After, some blood peripancreatic and epididymal fat samples were taken, and then, the heart was removed. Finally, the animals were euthanized by cervical dislocation.

### 2.2. Biochemical Measurements

Peripheral venous blood samples were used to quantify glucose, insulin, triglycerides, and total cholesterol using standard laboratory techniques in 8-hour-fasted rats [26]. The insulin resistance (IR) was quantified via the homeostasis model assessment of HOMA IR = serum insulin (uUI/ml) ∗ (blood glucose (mmol/L)/22.5 [27, 28].

### 2.3. Cardiac Function

The ECG was performed on anesthetized (0.5 mg/0.2 mg ketamine-xylazine/kg weight) rats. Bipolar ECGs were recorded using subcutaneous needle electrodes with Lead-I configuration; that signal was amplified in 700x and digitalized and captured to 10 kHz frequency for thirty minutes [25]. Data was stored in a personal computer and analyzed off-line, using Clampfit (molecular devices). All rats were continuously monitored to guarantee the right ventilation and temperature.

### 2.4. Ventricular Function

The heart was quickly removed and placed in a retrograde perfusion system, with Tyrode's solution at 36°C to wash the heart. In addition, excitation-contraction coupling was measured in the ventricular left and right papillary muscle isolates placed in a chamber to record simultaneously contraction force and action potential perfused with Tyrode's solution at 36°C, gassed with carbogen.

According to Frank-Starling's law [29], the contraction force was performed in the papillary muscles at Maxime longitude (Lmax). The action potential was recorded using the sharp microelectrodes of borosilicate (filled with 3 M KCl with a resistance of 25–35 M*Ω*). The signal was amplified with a WPI Duo 776 electrometer, digitalized (SCB-68 Quick references label, National Instruments), and analyzed using ClampFit (molecular devices) and Origin 7.0 (Southampton). The characterization of the action potential was measured with amplitude and the APD at 30 and 90% of the repolarization. The excitation-contraction coupling phenom was measured, using the time between the maximum voltage of the action potential and the maximum force of the contraction, including the delay between the start of the action potential with the start of the force of contraction [30].

### 2.5. Isolated Ventricular Cells from the Heart

Rat heart was cannulated through the aorta and perfused, according to the Langendorff method. Consequently, the heart was perfused in a recirculation mode for 8 minutes with collagenase, 28 mg/50 ml (Sigma Aldrich) and protease 1 mg/50 ml in solution in a Ca^2+^ free buffer. Finally, papillary muscle was mechanically dissociated in Kraft-Brüeh (KB) solution [31].

### 2.6. Alterations in Ventricular Electrical Activity

The patch-clamp technique was applied in the whole-cell configuration to record total currents; the patch pipettes had a resistance between 2 and 4 MOhm. The signal was captured at 5.4 kHz and amplified, digitalized (Heka), and stored on a personal computer. The cardiomyocytes were placed in a perfusion chamber in an inverted microscope (Nikon). Only Ca^2+^ tolerant rod-shaped right and left papillary cells were selected for this study.

### 2.7. Potassium Currents

The potassium current (I k) was elicited from a holding potential of -80 mV by a square voltage pulse of -40 mv at 5 ms, and depolarizing pulses to membrane potential from -40 mV to 50 mV for 500 ms were applied with 10 mV increments at 2 seconds intervals [31].

### 2.8. Solutions

The Clampex program of the pClamp software controlled the current- and voltage-clamp experimental protocols. The solution using during voltage clamp experiments was a normal external solution (in mM NaCl, 136; KCl, 5.4; MgCl_2_, 1; HEPES-Na, 10; CaCl_2_, 1.8; dextrose, 11; pH adjusted to 7.4 with NaOH). The potassium currents were recorded with extracellular solution that containing (in mM) choline chloride, 136; MgCl_2_, 1; HEPES-K+, 4; HEPES, 6; N-Methyl-D-glucamine, 6; CaCl_2_, 0.1; CoCl_2_, 0.5; and dextrose, 11; and it was adjusted with KOH to pH 7.4. The KB solution Isenberg & Klockner 1982 is the following composition (in mM): taurine, 10; glutamic acid, 70; creatine, 0.5; succinic acid, 5; dextrose, 10; KH2PO_4_, 10; KCl, 20; HEPES-K+, 10; and EGTA-K+, to adjust the pH to 7.4 with KOH [31]. The solutions to patch pipetes or internal solutions had the following composition (mM): 80 potassium aspartate, 10 KH_2_PO_4_, 1 MgSO_4_,7 H_2_O, 40 KCl, 10 HEPES, 10 EGTA, 3 Na_2_ATP, and 0.2 NaGTP; pH was adjusted to 7.3 with KOH.

### 2.9. Cardiac Histopathologic Alterations

The ventricular muscle was embedded in paraffin for 5 *μ*m, and cuts were done from the base to apex. After deparaffinization, the sections were stained with hematoxylin-eosin. The tissue samples were examined under a 40x microscope, and it was semiquantitatively analyzed with the Image J program.

### 2.10. Heart Rhythm Alterations

For analysis of the heart rate variability, we used the Poincare plot, in which the time series of ECG Intervals were plotted against the next value in a Cartesian coordinate system. In this research, the Poincare plots were constructed with RR and QT intervals of the recorded ECG, of all rat groups. The heart rate variability was quantified using the means of parameters SD1, SD2, and SD1/SD1 in each condition research [19].

### 2.11. Statistical Analysis

All data were analyzed using descriptive statics and expressed as means ± SD (Origin Pro 2017 and Clampfit 10.7). If the results presented a normal distribution and equal variance, Student's unpaired *t*-test or one-way ANOVA was used by Dunnett's post hoc test. If the data presented a no-normal distribution or equal variance, the Mann–Whitney U test or Kruskal–Wallis test was performed. It was assumed to be a significant change if *p* < 0.05.

## 3. Results

### 3.1. Animal Model with Different Alterations in the Obesity of Metabolic Syndrome Profile

In the rodents, the high-sucrose diet by 24 weeks, developed MetS with three of the five metabolic (see Table 1) alterations as described by literature. In this model, seventy percent of those rats (*n* = 14) showed MetS with only a 2% body weight gain that is NOMetS, and this subgroup had the same abdominal circumference compared to the control group. However, these rats had 70% more epididymal fat than the control group. The remaining rats with MetS (*n* = 6) showed a 40% body-weight gain, and OMetS were named as subgroups.

In the abdominal circumference and epididymis fat, a significant increase of 18% was detected in the NOMetS subgroup, while the value in the OMetS subgroup was 300%, compared to control. All Wistar rats with MetS presented alterations of lipid metabolism. Although, the data of NOMetS rats showed a significant increase in triglycerides (TG) levels compared to OMetS, 153 mg/dl and 116 mg/dl, as well as in c-LDL 47 mg/dl and 23 mg/dl respectively, see Table 1.

### 3.2. Effect of Obesity Profile on Heart Rhythm

**Figure 1:**
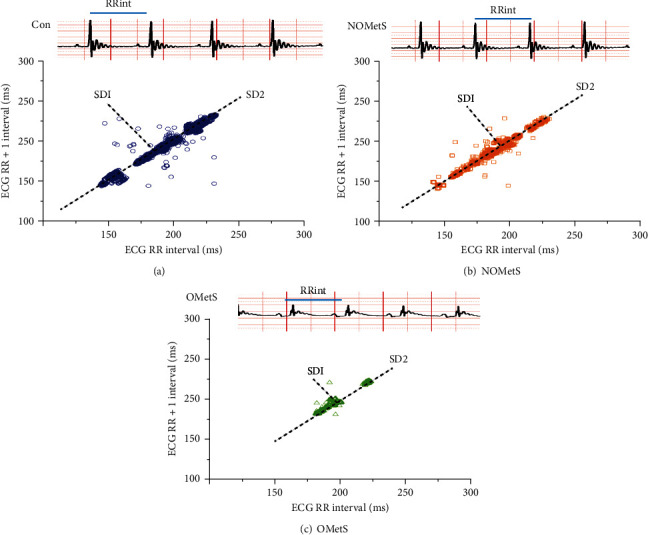


**Figure 2:**
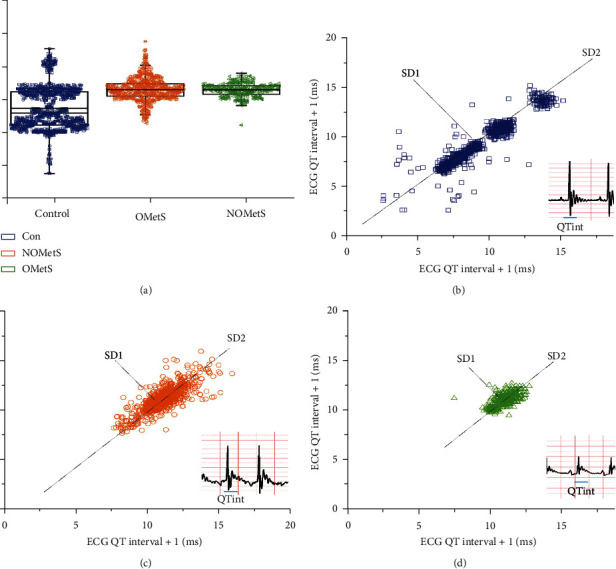


The OMetS animal model presented a decrease of 308 ± 0.9 bpm in heart rate, while in the NOMetS subgroup, there was an increase of 330 ± 1 bpm compared to the control group which had 317 ± 0.5 bpm. Interestingly, the effect of obesity profile on the electrocardiogram record, in both cases NOMetS and OMetS, was observed as in long QT syndrome; hence, the QTc values were longer 0.66 ± 0.05 and 0.66 ± 0.07 ms, respectively, than the control group, 0.55 ± 0.1 ms (Figures 1 and 2).

### 3.3. The Heart Rate Variability by Obesity

The cardiac health was evaluated with heart rate variability using Poincare plots, and as expected, the control group revealed an ellipse shape behavior (Figure 1(a)), with a variability of SD1 = 1.03, SD2 = 1.31, and index SD1/SD2 = 0.79 see Table 2. Together, in the data of heart rate variability of NOMetS rats, the ellipse shape is like the control group. Above the line identity of SD2 are registered the 80% of RR intervals, and SD1/SD2 index is decreased (Figure 1(b)). In the plot of OMetS subgroup, a decrease was observed in the heart rate variability in the SD1, SD2, and index SD1/SD2 biomarkers (see Figure 1(c)) compared with the control set (Table 2).

The beat-to-beat variations are also extremely sensitive to small fluctuations in several levels (Figure 2(a)). Thus, the variability in the QT intervals was quantified, which tends to behave in typical ellipse shape in the Poincare plots of RR interval (Figures 2(b)–2(d)). The quantitative analysis of the QT interval showed changes in the three biomarkers, only in the OMetS subgroup (Table 2). The variability of the NOMetS subgroup had an increase in SD1 = 1.8, SD2 = 2.8, and the SD1/SD2 = 0.75 index (see Figure 2(c) and Table 2), meanwhile in the OMetS heart rate variability also increased, but not showing significant differences between aggregate sets (see Figure 2(d)).

### 3.4. Alterations in Ventricular Function Related to Obesity

**Figure 3:**
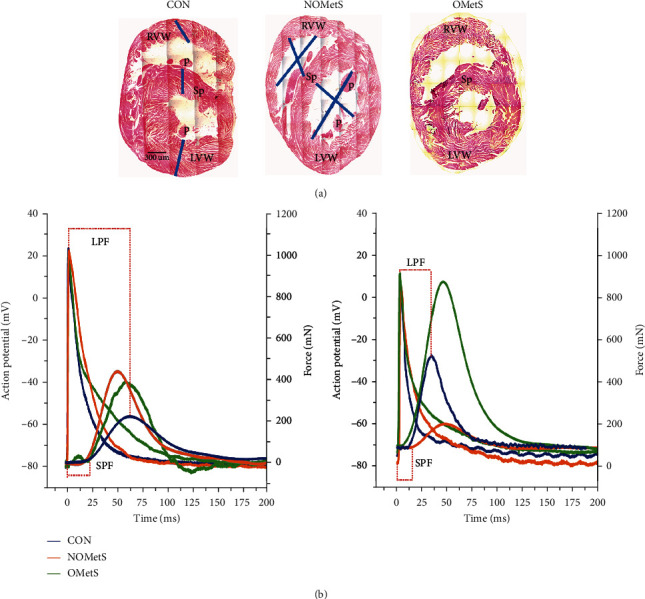


Our results indicated that obesity profile altered the ventricular function, which was measured with excitation-contraction (E-C) coupling phenom. In the OMetS subgroup, the force contraction and action potential duration (APD) were higher in the left papillary ventricle than the right one. Consequently, E-C coupling was the desired outcome for ventricular function in the control rats (see Figure 3(b), Table 3). The NOMetS model showed alterations in the electrical and mechanical mechanisms of the E-C coupling. The contraction force increased 66 percent in the right and decreased 57 percent in the left papillary muscle while in relationship with the APD had an increased 61 percent and decreased 40 percent, respectively.

However, the left papillary muscle showed a decoupling which was related to the reduction in the latency period (LPF) and the start period (SPF) of the contraction force ( see Figure 3(b) and Table 3). The OMetS subgroup also presented an increase in the mechanisms of E-C coupling, showing a decoupled left papillary muscle of 30% and 40% in LPF and SPF, respectively, (Figures 3(a) and 3(b) and Table 3). The force was almost 2-fold than the control group.

High-sucrose diet-induced obesity is associated with hypertrophic cardiomyopathy [32]. The Hematoxylin-eosin staining (Figure 3(a)) suggests a ventricular hypertrophy in the NOMetS subgroup. The ventricles of the heart had a perimeter of 7.5 ± 0.5  mm, 6.5 ± 0.3  mm, and 4.6 ± 0.3  mm for NOMetS, OMetS, and control, respectively (Figure 3(a)). The MetS do not alter the ventricular-wall size, and the data showed 3.3 ± 1, 2.8 ± 0.8 and 3.1 mm for NOMetS, OMetS, and control. Of note, there was no significant difference in heart weight, and the data were 2.1, 2.3 and 1.98 g for NOMetS, OMets, and control, respectively.

### 3.5. The Obesity Profile Is Associated with Alterations in the Activity of Potassium Currents

**Figure 4:**
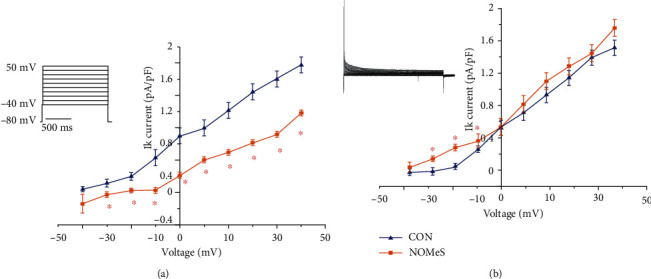


The increase in APD of ventricles in NOMetS rats allowed indirectly induced changes in the potassium total currents. For this reason, we recorded potassium currents in isolated myocytes and the data showed a decrease in the amplitude of potassium current (Ito) in the right papillary muscle [33] (Figure 4(a)). Also, we measured the potassium current in myocytes of left papillary muscle; these currents showed an increase at only negative voltages (Figure 4(b)). Furthermore, the amplitude of each current component, obtained from the fitting, was normalized to cell capacitance to compare current densities from cells of different sizes. The NOMetS subgroup did not affect the resting membrane potential of ventricular papillary muscle (see Figure 4).

## 4. Discussion and Conclusions

This study proposed to identify cardiac electrical disease by electrical and metabolic biomarkers, using heart rate variability of RR, QT intervals, and blood plasma biochemistry to improve the diagnostic and prognosis of cardiometabolic diseases. Recent evidence suggests that genetic and environmental factors contribute to the MetS development; such factors are high-carbohydrates, high-fat diets, and lack of physical activity [24]. These factors promote insulin resistance, impaired fasting glucose, alteration of lipids metabolism, and a chronic inflammatory state and visceral obesity [34]. In this study, *Wistar* rats are not genetically susceptible to develop obesity [24]; though, a high-sucrose diet in drinking water like environmental factors produced MetS with resistance to insulin and dyslipidemia and impaired fasting glucose after 2 months of treatment [24]. In this model, after this twenty-eight-week high-sucrose diet, the rats presented MetS with and without obesity [35]. During a period of high-sucrose diet intake in the animal model, the liver tissue converted glucose into fatty acids and stored them in the adipose tissue [8, 36]. Consequently, in this study, both subgroups had increased in the distribution of epididymal fat weight by 0.7 and 2.5 times for NOMetS and OMetS, respectively (see Table 1). The NOMets rats were showing insulin resistance, and this data indicated in the rats a behavior of excess nutrients in their metabolism. This suggests an imbalance in the storage and synthesis of lipids in the liver, and the TG concentration in plasma is higher than control and OMetS [37]. Also, the NOMetS group had c-LDL and c-HDL plasma concentration higher than OMetS, and both proteins respond to the mechanism of high blood lipid metabolism and low reservoir in fat tissue [5]. These results explain that the rats with obesity only had the mechanism to reverse cholesterol transport [5].

The excess of fatty acids in plasma and insulin resistance has been proposed as a key driver for accumulation in obese individuals [2, 38]. In the MetS, the overweight model or OMetS under the higher-sucrose diet was enough to have an excess of triglycerides in plasma but c-HDL and insulin, similarly, in the control set. Furthermore, this model had alterations in HOMA-IR index [27] and the peripancreatic and epididymal fat increasing in 3 times and 85%, respectively, more than the control group. In general, obesity has been associated with the left ventricular dysfunction and cardiomyopathies by ventricular hypertrophy [39, 40].

However, the OMetS subjects only showed the electromechanical alterations in the function of both ventricles due to obesity. The outcomes revealed long QT syndrome related to alterations in the duration of AP ventricular and changes in the dispersion in RR. Also, the OMetS subgroup had alterations in the function of both ventricles (Figures 3(a) and 3(b). Table 3) and bradycardia, and the ventricle force augmented without alterations in ventricular morphology (Figure 3(b)).

On the other hand, the NOMetS subgroup presented syndrome long-QT with an increase in APD 90% in both chambers even without obesity, and it is worth mentioning that the clinical biomarkers of dyslipidemia and obesity were higher than that of the obese subgroup. The beginning of the QRS complex has been defined by the onset of ventricular electrical activity; the NOMetS. Also, this subgroup showed dispersion in QT intervals. This is related to susceptibility to reentry ventricular tachyarrhythmias and, revealed dysfunction in both ventricles, the contraction force was reduced in left papillary muscle, and the right papillary was enhanced, these alterations are caused by the changes to the electrical activity. Furthermore, the dispersion in electrical activity, at the start, the repolarization of action potential and the electro-mechanical changes allowed us to provoke the long QT syndrome in these animals [41].

On the other hand, the excitation-contraction decoupling was associated with hypertrophy [42]. In this model, the animals were fed with high sucrose diet and only the NOMetS subgroup presented eccentric hypertrophy associated with over ventricular dysfunction and fat tissue (see Table 1). These were related to the release of catecholamines in the heart [43]. Additionally, in NOMetS subgroup, tachycardia was observed, which is related to a parasympathetic innervation increase of pacemaker tissue [25].

The events that occur in E-C coupling of cardiac muscle depend on calcium concentration and the duration of action potential [44]. The lethal arrhythmia as the long QTc syndrome is correlated with APD prolongation, and the main cause is the reduction in the densities of K repolarizing currents [45]. In the NOMetS animals, the alterations in the start and latency periods are presented, and an increase of APD associated with IK current decrease in the right ventricle [46] (see Figure 4).

In humans, the common central mechanisms modulated by both sympathetic and parasympathetic cardiovascular modulation were measured with HRV [47]. This biomarker is used to prognostic and diagnose health of the heart in metabolic diseases [48]. In this work, the HRV was impaired according to the levels of body fat, and the OMetS animals showed a decreased standard deviation in both mechanisms of modulation of the autonomic nervous system. The NOMetS animals presented an increase in the variability, only in QT intervals and the participation of the parasympathetic system (Figure 1(b)), raising the likelihood of lethal arrhythmias as long QT (Figure 2(a)), and the increase of modulation by the sympathetic and parasympathetic system on electrical properties of the ventricle (Figure 2(c)).

In this study, the c-HDL is high in both subgroups in which the protection was conferred to cardiovascular diseases [49] and attached to atherosclerosis [26]. However, both subgroups had high probability for lethal arrhythmias due to long QT syndrome and dyslipidemias (Table 1).

Additionally, the ventricular ejection force was measured with strain papillary muscle [42]; this data allowed indirectly to quantify the peripheral blood flow and ventricular chamber volumes [50]. The outcomes of the E-C coupling suggest that the left and right ventricles reflected alterations like cardiomyopathy in both MetS subgroups. The MetS with obesity did not alter morphologically the ventricles (Figure 3(a) and Table 3). Meanwhile, the H-E staining data revealed that the nonobese group exhibited a dilated cardiomyopathy [51]. The data showed the ventricular arrhythmias were produced by the MetS.

Finally, the type of ventricular arrhythmia depends on whether obesity is present or not. The outcomes suggest that the MetS without obesity promotes a poor prognosis of cardiomyopathies, and these alterations could be measured for prognostic and diagnostic purposes for the heart rate variability of ECG. The OMetS rats presented alterations of HRV with decreasing of asymmetry Poincare of RR interval, associated with increased contraction force in both ventricles and electrical alterations. However, the NOMetS animals had higher alterations in the metabolism, and the ventricular electrical activity are strongly correlated to long-QT syndrome, QT variability, and hypertrophic ventricular.

### 4.1. Statement Experimental Protocols

All animal procedures were performed in accordance with “International Guiding Principles for Biomedical Research Involving Animals”, Council for International Organization of Medical science 2010. The protocol was approved by the ethics committee (CICCUAL-PROYECTO-00365) of the Benemérita Universidad Autónoma de Puebla.

## Figures and Tables

**Table 1:** Metabolic Characterization of animal model.

	Control (*n* = 20)	NOMeS (*n* = 14)	OMeS (*n* = 6)
Morphometric parameters			
Length (cm)	25.6 ± 0.2	25.7 ± 0.2	27.6 ± 0.5^∗^
Waist (cm)	23.4 ± 0.7	24 ± 0.7	27.8 ± 0.6^∗^
Weight (g)	518 ± 12	529 ± 5	725 ± 22^∗^^†^
Epididymal fat (g)	4.6 ± 0.4	7.9 ± 0.5^∗^	16.3 ± 2^∗^^†^
Peripancreatic fat (g)	1.97 ± 0.5	2.1 ± 0.4	3.9 ± 0.8^∗^^†^
BMI (kg·m^−3^)	31 ± 0.6	31 ± 0.6	36 ± 0.1^∗^
Clinical biochemistry values			
Glucose (mg·dl^−1^)	90 ± 2	104 ± 3^∗^	112 ± 7^∗^
Urea	39 ± 10.6	29 ± 9^∗^	33 ± 8^∗^
Triglycerides (mg·dl^−1^)	75 ± 7	153 ± 33^∗^	116 ± 22^∗^
Insulin (pM·l^−1^)	26 ± 0.02	27 ± 0.03	22 ± 0.04
HDL-c (mg·dl^−1^)	15 ± 2	31 ± 7^∗^	23 ± 5^∗^^†^
LDL-c (mg·dl^−1^)	21 ± 4	47 ± 14^∗^	23 ± 5^†^
HOMA-IR	0.8	1^∗^	0.9^∗^

Mean ± SD;*p* ≤ 0.05, ^∗^vs control, ^†^vs NOMetS.

**Table 2:** Heart rate variability.

	CON (*n* = 20)	NOMetS (*n* = 14)	OMetS (*n* = 6)
ECG RR interval			
SD1	1.03	1.04	0.51^∗^^†^
SD2	1.31	1.53	0.74^∗^^†^
SD1/SD2	0.79	0.68^∗^	0.69^∗^
ECG QT interval			
SD1	1.14	1.8^∗^	1.5
SD2	1.5	2.8^∗^	1.8
SD1/SD2	0.88	0.75^∗^	0.89

Mean ± SD;*p* ≤ 0.05, ^∗^vs control, ^†^vs NOMetS.

**Table 3:** Electrical and mechanical ventricular function.

	Control (*n* = 20)		NOMetS (*n* = 14)		OMetS (*n* = 6)	
	RPM	LPM	RPM	LPM	RPM	LPM
Amplitude (mV)	96 ± 2.4	87 ± 2	97 ± 1.6	91 ± 9	93 ± 2	79 ± 2^∗^
APD 30% (ms)	6 ± 0.4	8 ± 0.8	6 ± 0.4	5.8 ± 0.4	7 ± 0.3^∗^	7.4 ± 0.5
APD 90% (ms)	41 ± 1.8	38 ± 3	67 ± 4.4^∗^	57 ± 2^∗^	47 ± 2.7^∗^^†^	70 ± 5.2^∗^
LPF (ms)	57	73	56	50^∗^	46^∗^	49^∗^
SPF (ms)	14	17	12	15^∗^	12	10^∗^
Force (mN)	311	477^†^	519^∗^	205^∗^	615^∗^	839^∗^^†^

APD: action potential duration; LPM: left papillary muscle; RPM: right papillary muscle; LPF: latency period force; SPF: start period force. Mean ± SD;*p* ≤ 0.05, ^∗^vs control, ^†^vs NOMetS.

## Data Availability

The data sets used and/or analyzed during the current study are available from all authors on reasonable request.
